# Risk factors of adverse birth outcomes among a cohort of pregnant women in Coastal Kenya, 2017–2019

**DOI:** 10.1186/s12884-024-06320-6

**Published:** 2024-02-12

**Authors:** Harriet Mirieri, Ruth Nduati, Jeanette Dawa, Lydia Okutoyi, Eric Osoro, Cyrus Mugo, Dalton Wamalwa, Hafsa Jin, Dufton Mwaengo, Nancy Otieno, Doris Marwanga, Mufida Shabibi, Peninah Munyua, John Kinuthia, Erin Clancey, Marc-Alain Widdowson, M. Kariuki Njenga, Jennifer R. Verani, Irene Inwani

**Affiliations:** 1 Washington State University (WSU) Global Health Kenya, Nairobi, Kenya; 2 Department of Paediatrics and Child Health, University of Nairobi, Nairobi, Kenya; 3 Department of Health Care Quality, Kenyatta National Hospital, Nairobi, Kenya; 4 Paul G. Allen School of Global Health, Washington State University (WSU), Pullman, USA; 5 Department of Research and Programs, Kenyatta National Hospital, Nairobi, Kenya; 6 Coast General Teaching and Referral Hospital, Mombasa, Kenya; 7 University of Nairobi Institute of Tropical and Infectious Diseases, Nairobi, Kenya; 8 Centre for Global Health Research, Kenya Medical Research Institute, Kisumu, Kenya; 9 Port Reitz Sub County Hospital, Mombasa, Kenya; 10 Division of Global Health Protection, Centers for Disease Control and Prevention, Nairobi, Kenya; 11 Department of Paediatrics, Kenyatta National Hospital, Nairobi, Kenya; 12 Institute of Tropical Medicine, Antwerp, Belgium

**Keywords:** Adverse birth outcomes, Preterm birth, Small for gestational age, Stillbirth, Miscarriage, Microcephaly

## Abstract

**Introduction:**

Adverse birth outcomes particularly preterm births and congenital anomalies, are the leading causes of infant mortality globally, and the burden is highest in developing countries. We set out to determine the frequency of adverse birth outcomes and the risk factors associated with such outcomes in a cohort of pregnant women in Kenya.

**Methods:**

From October 2017 to July 2019, pregnant women < 28 weeks gestation were enrolled and followed up until delivery in three hospitals in coastal Kenya. Newborns were examined at delivery. Among women with birth outcome data, we assessed the frequency of congenital anomalies defined as gastroschisis, umbilical hernia, limb abnormalities and Trisomy 21, and adverse birth outcomes, defined as either stillbirth, miscarriage, preterm birth, small for gestational age, or microcephaly. We used log-binomial regression to identify maternal characteristics associated with the presence of at least one adverse outcome.

**Results:**

Among the 2312 women enrolled, 1916 (82.9%) had birth outcome data. Overall, 402/1916 (20.9%; 95% confidence interval (CI): 19.1–22.8) pregnancies had adverse birth outcomes. Specifically, 66/1916 (3.4%; 95% CI: 2.7–4.4) were stillbirths, 34/1916 (1.8%; 95% CI: 1.2–2.4) were miscarriages and 23/1816 (1.2%; 95% CI: 0.8–1.9) had congenital anomalies. Among the participants with anthropometric measurements data, 142/1200 (11.8%; 95% CI: 10.1 − 13.8) were small for gestational age and among the participants with ultrasound records, 143/1711 (8.4%; 95% CI: 7.1–9.8) were preterm. Febrile illnesses in current pregnancy (adjusted risk ratio (aRR): 1.7; 95% CI: 1.1–2.8), a history of poor birth outcomes in prior pregnancy (aRR: 1.8; 95% CI: 1.3–2.4) and high blood pressure in pregnancy (aRR: 3.9, 95% CI: (1.7–9.2) were independently associated with adverse birth outcomes in a model that included age, education, human immunodeficiency virus status and high blood pressure at enrolment.

**Conclusion:**

We found similar rates of overall adverse birth outcomes, congenital anomalies, and small for gestational age but higher rates of stillbirths and lower rates of prematurity compared to the rates that have been reported in the sub-Saharan Africa region. However, the rates of adverse birth outcomes in this study were comparable to other studies conducted in Kenya. Febrile illnesses during the current pregnancy, previous history of poor birth outcomes and high blood pressure in pregnancy are predictive of an increased risk of adverse birth outcomes.

**Supplementary Information:**

The online version contains supplementary material available at 10.1186/s12884-024-06320-6.

## Introduction

Despite considerable efforts to improve coverage of maternal, neonatal, and child health services, neonatal mortality remains a significant public health problem, particularly in developing countries, and the leading causes include congenital anomalies and preterm births [1–3]. Similarly, small for gestational age infants are at greater risk of neonatal morbidity and mortality [4]. Globally, of all the live births, 27% are small for gestational age (SGA) [5], 1.3% are stillbirths [6], 10.6% of are preterm [7], and 2.2% of newborns have structural congenital anomalies [8]. The burden of adverse birth outcomes is relatively high in sub-Saharan Africa, where of all live births, 25.5% are SGA [5], 12.3% are preterm [9], 2.1% are stillbirths [6, 10], and 2.4% of newborns have a congenital anomaly [11]. Rates are equally high in Kenya, where 1.9% of the live births are stillbirths [12], the prevalence of congenital anomalies is 1.9% [13] and up to 12% of all live births are small for gestational age [14].

Stillbirths and miscarriages have a significant negative economic and emotional impact on the affected families [8, 14] and children who are born prematurely have higher rates of sensory deficits, respiratory illnesses, and experience developmental delays as well as learning disabilities [1, 15]. In addition, infants born small for gestational age have an increased risk of mortality as a result of infections and neurologic diseases [4] and may experience cognitive impairments and developmental delays in childhood [16]. Similarly, congenital anomalies contribute to long-term disability and social stigma which negatively impacts individuals and their families [8].

The aetiologies of adverse birth outcomes are multifactorial and vary across settings. Studies have shown that context specific sociodemographic factors, obstetric factors, comorbidities, and maternal clinical conditions, including nutritional status, and health service utilization behaviour, are predictors of adverse birth outcomes [16–20]. In Kenya, initiatives aimed at improving maternal child health have been implemented; services available at public health facilities include free antenatal care, antiretroviral medicines for pregnant women living with human immunodeficiency virus (HIV), syphilis screening, provision of insecticide-treated nets, administration of intermittent preventive treatment for malaria, micronutrients supplementation, skilled delivery and immediate postnatal care services [21]. However, despite these strategies, the burden of adverse birth outcomes still remains high [22]. This may indicate a complex interplay of risk factors that vary by context. It is, therefore, critical to periodically gather local evidence to improve the effectiveness of existing maternal and child health programmes and guide prevention efforts.

A previous population-based survey in Kenya identified very young (< 20 years) or advanced maternal age (40–49 years), shorter birth intervals, and maternal nutritional status as the potential determinants of adverse birth outcomes [14]. Other cross-sectional studies on poor birth outcomes in Kenya have reported lower education level, preexisting medical conditions, multiparity and history of adverse birth outcomes as potential risk factors for poor birth outcomes [22–24]. However, cross-sectional surveys have significant limitations, including recall bias and the inability to establish the temporal sequence of the exposure and outcome. This analysis, based on a prospective cohort study of pregnant women in Kenya, aimed at establishing the magnitude and factors associated with adverse birth outcomes.

## Methods

### Study design

This was a secondary analysis of a prospective cohort study that was conducted from October 2017 to August 2019 to estimate the incidence of Zika virus infection among pregnant women in the coastal county of Mombasa, Kenya. This cohort has been described previously [25].

### Study population

Briefly, study participants aged ≥ 15 years and below 28 weeks gestation that were planning to deliver at one of the study sites were recruited from the antenatal care (ANC) clinics after obtaining informed consent. In Mombasa county, 94% of the deliveries occur in health care facilities [26]. Women who had ectopic or molar pregnancies, incarcerated women, and those that were participating in trials involving experimental drugs and devices were excluded from the study. A total of 2312 pregnant women attending ANC clinics in three health facilities - one private facility (Bomu Hospital) and two public facilities (Coast General Teaching and Referral Hospital and Port Reitz Sub County Hospital) were enrolled in the parent study. Our study included all the participants that were enrolled in that cohort.

### Study procedures

#### Recruitment

Study staff directly recruited pregnant women attending the ANC clinic for either for confirmation of pregnancy or for prenatal care. Some participants were referred by providers from ANC clinic for recruitment. The participants were consented and a standard questionnaire was administered at enrolment. Data on sociodemographic characteristics, lifestyle factors, nutritional exposures, environmental exposures, and obstetric and medical history were collected. We abstracted data on haemoglobin levels, HIV, and syphilis status from the mother and child booklet. We calculated gestation age based on ultrasound imaging that was conducted at enrolment by a trained study sonographer or if unavailable, the date of the last menstrual period (LMP).

#### Follow-up

We followed up with participants during monthly clinic visits and administered a routine questionnaire at each visit until delivery. WHO recommends a total of eight ANC clinic visits during pregnancy [27] and the study visits were scheduled to coincide with these clinic visits. If the participants developed a fever in between visits, they were asked to come for the study visit for the collection of data, and samples were collected (urine and blood) before referral for routine clinical management. At each visit, the temperature recording was taken or abstracted from patient records. After delivery, a questionnaire was completed to collect information on the delivery process, birth outcomes, and complications.

#### Infant assessment

Study staff examined new-borns for vital signs, congenital anomalies and took anthropometric measurements within 24 h of birth. We measured the occipitofrontal head circumference thrice to the nearest 0.1 cm and the largest measurement recorded [28]. The infant’s weight was taken using the Salter digital infant scale, and the weight was recorded to the nearest gram.

Data from the structured questionnaires recorded stillbirths and miscarriages, data on prematurity were based on the estimated gestational age at delivery based on ultrasound, where the participants with missing ultrasound records were excluded while data on congenital anomalies were based on the physical examination. In contrast, the data for small for gestational age and microcephaly were based on the anthropometric measurements. Data were collected using REDCap software [29] and structured questionnaires were administered by trained study staff.

Participants were considered lost to follow up if the study staff could not establish contact with them and the delivery outcome was not recorded. In addition, participants who were no longer willing to participate in the study were withdrawn from the study.

### Study definitions

An adverse birth outcome was defined as either a miscarriage (pregnancy loss at < 22 completed weeks of gestation), stillbirth (pregnancy loss at ≥ 22 completed weeks of gestation), preterm (< 37 weeks gestation based on ultrasound), small for gestational age (birth weight of less than 10th percentile (Z score of < -1.28) for gestational age) based on the INTERGROWTH-21st charts and/or microcephaly (head circumference < 2 standard deviations (SD) below the mean for sex and gestational age using the INTERGROWTH-21st charts) [30]. We used a composite adverse birth outcome due to the possible overlap of risk factors of the four outcomes [31, 32] and to maximize the statistical power of the study [33]. Congenital anomalies were defined as gastroschisis, umbilical hernia, limb abnormalities orTrisomy 21. These congenital anomalies were excluded in the analysis of risk factors for adverse birth outcomes as the physiological processes that cause them to vary significantly from the other four and could therefore have different risk factors [34].

Other maternal variables of interest were defined as follows: chronic disease – reported history of asthma, epilepsy, diabetes or hypertension; anaemia – haemoglobin level < 10.5 g/dl; birth outcome – results of a pregnancy which were either a live birth, miscarriage or stillbirth; history of poor birth outcome – a history of stillbirth, miscarriage, premature birth or low birth weight baby in previous pregnancies among multiparous women; substance use – use of alcohol, tobacco or other recreational drugs, such as heroin, marijuana and cocaine; mid upper arm circumference – underweight (< 23 cm), normal weight (23–31 cm), and overweight (> 31 cm); short stature – height of < 145 cm [35] and febrile illness – documented or reported an axillary temperature of ≥ 38 °C at the clinic visit or within the preceding 7 days. Women were asked about fever episodes at enrolment, during the monthly clinic visits, through the biweekly phone call to the participants, or from participant-initiated calls to the study team.

### Ethical considerations

Approval to conduct the study was obtained from the Kenyatta National Hospital-University of Nairobi Ethics and Research Committee (P71/02/2017), Washington State University Institutional Review Board (No. 15,897), US Centers for Disease Control and Prevention (No. 7021) and Coast General Teaching and Referral Hospital Ethical Review Committee (ERC-CGH/MSc/VOL.1/32). Written informed consent was obtained from the participants prior to enrolling in the study.

### Statistical analysis

To describe the characteristics of the study population, descriptive statistics (medians, and interquartile ranges) were used to summarize each participant’s age, gestational age, and anthropometric measurements. Participants were grouped as either having birth outcome data or not, and comparisons between the two groups were made using univariable logistic analysis to determine the crude odds ratios of the observed differences and assess the degree of potential selection bias in the main analysis.

The prevalence of adverse birth outcomes during pregnancy was calculated among the participants with birth outcome data as the proportion of women who had an adverse birth outcome during pregnancy. We used generalized linear models (family = binomial and link = log) to fit the log-binomial regression models and calculate risk ratios. To select the best multivariable model to study risk factors, we first performed a univariable analysis between each predictor variable (sociodemographic, obstetric, lifestyle, and medical history) and the response variable (adverse birth outcome). Age was categorized into 3 groups: 15–19 years, 20 – ≤35 years, and 36 + years and assessed as a categorical variable to allow for comparison of the middle group to the youngest and oldest age groups which have been shown to have worse outcomes in previous studies. The multicollinearity of predictor variables with significant association with adverse birth outcomes in the univariable analysis were assessed using the variance inflation factor (VIF), and highly collinear variables dropped. Variables with a p ≤ 0.25 in the univariable analysis were then included in the multivariable model [36], where a backward stepwise approach was used to eliminate variables from the model if p > 0.05 while adjusting for other variables in the model and a new smaller model fitted. Each time we removed a variable we used a likelihood ratio test (LRT) to check if deleting a variable from the model had an impact on the model fit. Factors with a non-significant association with the outcome were eliminated from the model if their exclusion from the model did not result in a greater than 30% change in the coefficients of the remaining variables in the model. The last step in our model selection procedure used the Akaike Information Criterion (AIC) to select the best fit model with the lowest AIC.

We explored the interactions between all the variables in the final model. Adjusted risk ratios (aRR) and the corresponding 95% confidence intervals (CIs) and P-values using Wald statistics were used to estimate the strength of association between the predictors and the outcome. The Hosmer-Lemeshow goodness of fit test was used to assess model fitness with a p value of > 0.05 suggestive of a good fit [37]. Data were analysed using Stata version 13.0.

## Results

**Fig. 1:**
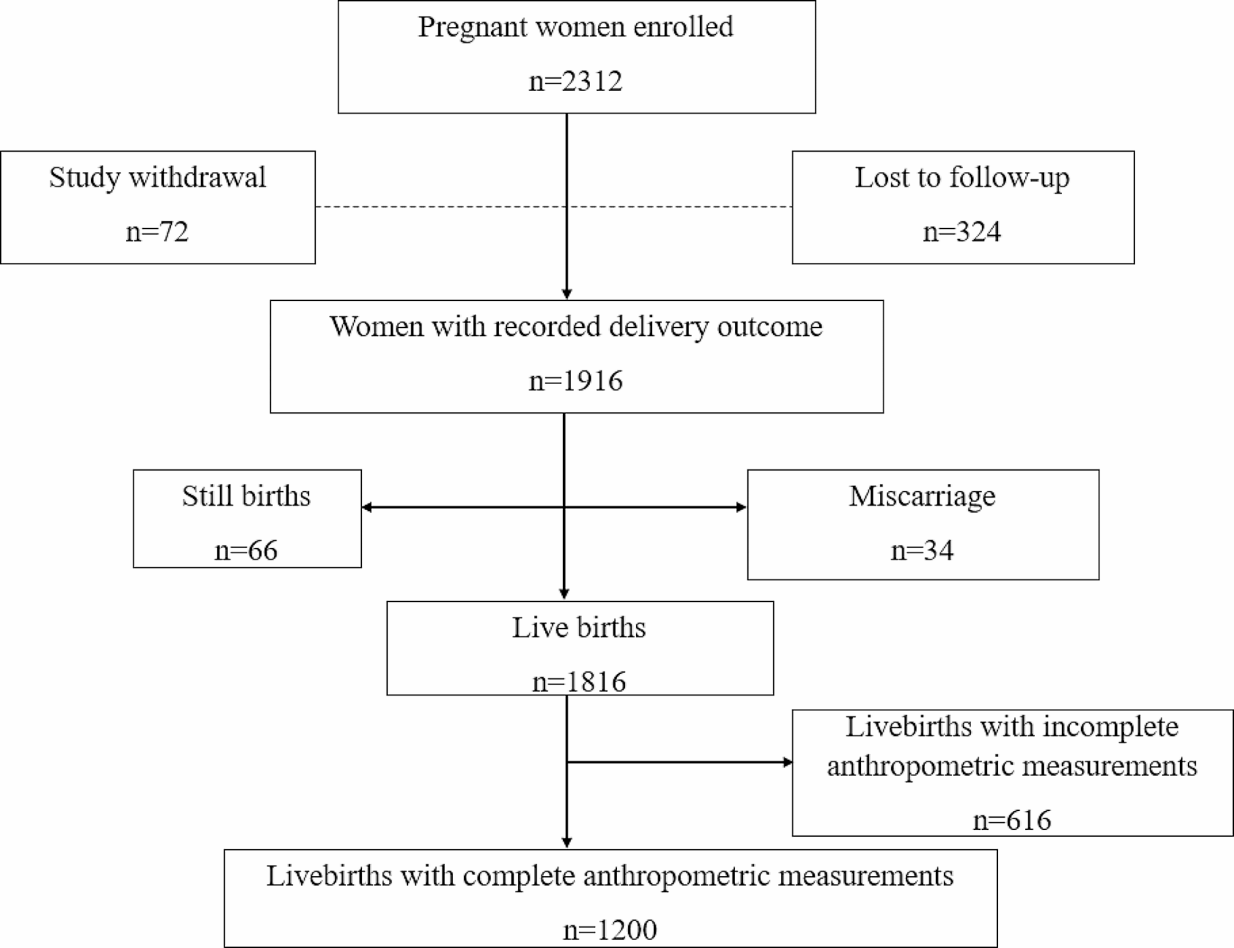
Flow diagram showing participant numbers during the study

A total of 2312 women were enrolled into the study. Among them, 1,916 (82.9%) had birth outcome data recorded, and 396 (17.1%) either withdrew from the study or were lost to follow up. More than half, 1200/1916 (62.6%) participants had complete newborn anthropometric measurements. About a third 616/1916 (32.1%) of the deliveries occurred outside the study sites hence did not have the compelete anthropometric measurements conducted at delivery (Fig. 1).

Women with birth outcome data were more likely to be older, multiparous, HIV positive and were likely to attend ANC clinics at public hospitals compared to women without outcome data (Supplementary Table 1).

### Characteristics of participants enrolled in the study

The median age of participants with birth outcome data was 28 years (interquartile range (IQR): 24–33), and the median gestation age at delivery was 39 weeks (IQR: 38–41).

The majority of the participants were married 1699/1916 (88.7%) while 538/1885 (28.5%) had attained college level education. A total of 308/1791 (17.2%) of the participants were HIV positive, and of these, the majority 278 (90.3%) were on highly active antiretroviral therapy. More than half of the participants (1367/1916, 71. 3%) were multiparous. Only 116/1905 (6.1%) of the participants had an underlying medical condition and of these, 59% had asthma, 31% had hypertension, 11% had chronic diabetes while 5% had epilepsy. A total of 22 participants had high blood pressure (22/1891, 1.2%) and 492/1916 (25.7%) had anaemia at enrolment. Among the participants with recorded syphilis status results, a total of 26/1728 (1.5%) tested positive for syphilis at enrolment. All participants had LMP recorded and 1711/1916 (89.3%) of the participants had ultrasound records.

A total of 88 of 1916 (4.6%; CI: 3.7–5.6) pregnant women reported at least one incident of documented or reported febrile illness during the current pregnancy. The median number of febrile illness episodes was 1 (range 1–3) with 4 (4.5%) women having more than one occurrence of febrile illness in the current pregnancy. The median age of the study participants with febrile illness was 27 years (IQR: 23 − 32). The median gestation age of these women during enrolment was 19.3 weeks (IQR: 15.0–24.5) while the median gestational age during febrile illness episodes was 28.2 weeks (IQR: 23.0–33.2). One third (31%) of the participants with fever reported a history of dengue or chikungunya diagnosis in the preceding one year. A total of 46 (52%) of the participants had ongoing fever at the time of enrolment and 34 (38.6%) of the patients that had fever sought medical care and of these, 8 (24%) had been admitted.

### Adverse birth outcomes among participants with birth outcome data

Overall, adverse birth outcomes were detected among 402/1916 (20.9%; 95% CI: 19.1–22.8) of study participants. Among the 1,916 womenthat had data on the birth outcomes of interest, 66 (3.4%; 95% CI: 2.7–4.4) had stillbirths, and 34 (1.8%; 95% CI: 1.2–2.4) had miscarriages. Out of the 1816 live births, 23 (1.3%; 95% CI: 0.8 − 1.9) congenital anomalies were recorded, of which 11 (0.6%) umbilical hernias, 10 (0.5%) limb deformities, one each gastroschisis andTrisomy 21. Among the 1711 participants with ultrasound records, 143 (8.4%; 95% CI: 7.1–9.8) were preterm. Among the 1236 participants with recorded head circumference, 11 (0.9%; 95% CI: 0.5–1.6) had microcephaly. A total of 1200 newborns had complete anthropometric measurements, and of these, 142 (11.8%; 95% CI: 10.1–13.8) were small for gestational age (Table 1).

**Table 1:** Summary of adverse birth outcomes and congenital anomalies among participants with delivery data

Adverse birth outcome	Number of pregnant women assessed (n)	n (%)
Stillbirths (≥ 22 weeks)	1916	66(3.4)
Miscarriage (< 22 weeks)	1916	34(1.8)
SGA	1200	142(11.8) ^a^
Microcephaly	1236	11(0.9) ^a^
Preterm birth (> 22 weeks, < 37 weeks)	1711	143(8.4%) ^a^
Congenital anomalies	1816	23(1.3%) ^b^
**Total**	1916	**402(20.9%)**

^a^5 births were both SGA and had microcephaly; 10 births were both preterm and SGA; 2 births were both preterm and had microcephaly, ^b^ Congenital anomalies: Gastroschisis − 1, umbilical hernia-11, limb abnormalities-10 and Trisomy 21 − 1

### Univariable analysis of factors associated with adverse birth outcomes among women

From the results of the univariable models, age, education, fever in the current pregnancy, HIV status, history of chronic illness, parity, high blood pressure at enrolment, and history of poor birth outcomes met the threshold for inclusion in the multivariable model at p ≤ 0.25 (Table 2).

**Table 2:** Univariable analysis of factors associated with adverse birth outcomes among women enrolled in the study (N = 1916)

Variable	Total n (%)	Had adverse birth outcome n (%)	No adverse birth outcome n (%)	RR (95% CI)	p- value
**Age N = 1905**					
Median (IQR)		28.5(24–33)	28.0(24–33)	1.0(0.9-1.0)	0.567
15–19	62(3.3)	17(4.5)	45(2.9)	1.4 (0.9–2.2)	0.138*
20–35	1592 (83.6)	291(77.4)	1244(81.4)	Ref	
36–46	251 (13.1)	68(18.1)	240 (15.7)	1.2(0.9–1.5)	
**Facility type N = 1916**					
Private	654 (34.1)	126(33.2)	528(34.3)	1.0(0.8–1.3)	0.684
Public	1262 (65.9)	253(66.7)	1009(65.6)	Ref	
**Education N = 1885**					0.107*
Primary	490 (26.0)	112(29.8)	378(25.0)	1.1(0.9–1.4)	
Secondary	857 (45.5)	155(41.3)	702(46.5)	0.9(0.7–1.1)	
College	538 (28.5)	108(28.8)	430(28.4)	Ref	
**Employment N = 1915**					
Employed	754 (39.4)	144(37.9)	610(39.7)	Ref	0.631
Self employed	285 (14.9)	62(16.4)	223(14.5)	1.1(0.9–1.4)	
Unemployed	876 (45.7)	173(45.7)	703(4.8)	1.0(0.8–1.5)	
**Marital status N = 1916**					
Married	1699 (88.7)	332(87.6)	1367(88.9)	1.0(0.7–1.4)	0.461
Single	217 (11.3)	47(12.4)	170(11.1)	Ref	
**History of chronic illness N = 1905**					
Yes	116 (6.1)	30 (8.0)	86 (5.6)	1.3(0.9–1.8)	0.084*
No	1789 (93.9)	345(92.0)	1444 (94.4)	Ref	
**Substance use N = 1916**					
Yes	75 (3.9)	18(4.7)	57(3.7)	1.2(0.8–1.9)	0.349
No	1841 (96.1)	361 (95.2)	1480 (96.3)	Ref	
**Anaemia at enrolment N = 1916**					
Yes	492 (25.7)	93(24.5)	399(25.9)	0.9(0.8–1.2)	0.572
No	1424 (74.3)	286(75.5)	1138 (74.0)	Ref	
**Folic acid intake N = 1916**					
Yes	494 (25.7)	57(22.9)	437(26.2)	0.8(0.6–1.1)	0.280
No	1422 (74.2)	191 (77.0)	1231 (73.8)	Ref	
**Short stature N = 1897**					
Yes	32(1.7)	7(1.9)	25(1.6)	1.1(0.6–2.1)	0.769
No	1865 (98.3)	369(98.1)	1496(98.4)	Ref	
**Syphilis status N = 1728**					0.371
Positive	26(1.5)	7(2.0)	19(1.4)	1.3(0.7–2.5)	
Negative	1702(98.5)	338(97.9)	1364(98.6)		
**MUAC of the pregnant women = 1479**					
Median (IQR)	28(26–31)	28(25.4–30.5)	28(26–31)	0.9(0.9-1.0)	0.942
Underweight	107(7.3)	26(9.0)	81(6.8)	1.2(0.8–1.7)	0.259
Normal	826(55.9)	165(57.3)	661(55.5)	Ref	
Overweight	546(36.9)	97(33.7)	449(37.7)	0.8(0.7–1.1)	
**HIV status N = 1791**					
Positive	308(17.2)	77(21.6)	231(16.1)	1.3 (1.0-1.7)	0.013*
Negative	1483 (82.8)	279 (78.4)	1204 (83.9)	Ref	
**On HAART N = 303**					
Yes	278 (90.3)	69(80.8)	209(92.1)	0.9(0.5–1.7)	0.725
No	25 (8.1)	7(9.2)	18(7.9)	Ref	
**Mode of delivery**					
Caesarean section	403 (26.3)	74 (23.9)	329 (27.0)	1.1 (0.9–1.4)	0.273
Vaginal delivery	1127 (73.7)	236 (76.1)	891 (73.0)	Ref	
**Number of previous pregnancies (Parity) N = 1916**					
0	549 (28.7)	120(31.7)	429(27.9)	1.2(0.9–1.4)	0.197*
1–3	1284 (67.0)	239(63.0)	1045(68.0)	1	
> 3	83 (4.3)	20(5.3)	63 (4.1)	1.2(0.9–1.9)	
**History of poor birth outcome among multiparous women N = 1367**					
Yes	508(37.1)	124(47.9)	384(34.7)	1.6(1.2–1.9)	< 0.001*
No	859 (62.8)	135 (52.1)	724 (65.3)	Ref	
**Gestation age at first ANC visit N = 1916**					
**Median gestation age at first ANC visit (IQR)**		17.7 (13.6–21.7)	17.8 (13.6–22.7)	0.9 (0.9-1.0)	0.699
**High blood pressure at enrolment N = 1913**					
Yes	22 (1.2)	10(2.6)	12(0.8)	2.3(1.4–3.7)	0.002*
No	1891 (98.9)	369 (97.4)	1522 (99.2)	Ref	
**Fever in current pregnancy N = 1915**					
Yes	88 (4.6)	25(6.6)	63(4.1)	1.7(1.1–2.6)	0.038*
No	1827 (95.4)	354(93.4)	1473 (95.9)	Ref	
**Consistent use of mosquito net N = 1916**					
Yes	1457(76.0)	291(76.9)	1166(75.9)	1.0(0.8–1.3)	0.707
No	459 (23.9)	88 (23.2)	371 (24.1)	Ref	

Abbreviations: antenatal care (ANC), human immunodeficiency virus (HIV), interquartile range (IQR), risk ratio (RR), adjusted risk ratio (aRR), confidence interval (CI), highly active antiretroviral therapy (HAART), mid upper arm circumference (MUAC), reference category(ref)

*Variables eligible for inclusion in the multivariable model (p ≤ 0.25)

### Multivariable analysis of factors associated with adverse birth outcomes among women enrolled in the study

Separate models were run for primiparous and multiparous women as primiparous women had a missing value for a history of poor birth outcomes which made them fall out of the multivariable model that included the history of poor birth outcomes variable. Parity was dropped due to collinearity with history of adverse birth outcome. The final model comprised of age, education, HIV status, history of poor birth outcomes, high blood pressure at enrolment, and fever in current pregnancy. The variable on history of poor birth outcomes was not included in the primiparous model. Of these variables, only fever in current pregnancy (aRR: 1.8; 95% CI: 1.3–2.4) and history of poor birth outcomes (aRR: 1.7; 95% CI: 1.1–2.8) were statistically significant in the multiparous model while high blood pressure at enrolment was significant in the primiparous model (aRR: 3.9, 95% CI: (1.7–9.2) (Table 3). None of the interaction terms between variables in the final models were statistically significant. The VIF score for predictor variables in the multivariable analysis was < 2.5 for all the variables. Both models had a good fit (Hosmer Lemeshow P = 0.67 and P = 0.963).

**Table 3:** Multivariable analysis of factors associated with adverse birth outcomes among multiparous and primiparous women enrolled in the study

	Multiparous women (N = 1367)		Primiparous women (N = 549)	
Variable	aRR (95% CI)	p- value	aRR (95% CI)	p- value
**Age**				
15–19	1.8 (0.5–5.9)	0.351	1.3 (0.8–2.1)	0.383
20–35	Ref		Ref	
36–46	1.2 (0.8–1.7)	0.293	0.9 (0.4–2.3)	0.923
**Education**				
Primary	1.0 (0.7–1.5)	0.919	1.2 (0.8–1.9)	0.388
Secondary	0.8 (0.6–1.2)	0.361	0.8 (0.5–1.1)	0.222
College	Ref		Ref	
**History of chronic illness**				
Yes	1.1 (0.7–1.9)	0.645	0.6 (0.2–2.1)	0.424
No	Ref		Ref	
**HIV status**				
Positive	1.2 (0.8–1.7)	0.403	1.5 (0.9–2.5)	0.290
Negative	Ref		Ref	
**High blood pressure at enrolment**				
Yes	1.7 (0.7–3.9)	0.257	3.9 (1.7–9.2)	**0.002**
No	Ref		Ref	
**Fever in current pregnancy**				
Yes	1.7 (1.1–2.8)	**0.042**	1.5 (0.7–3.1)	0.290
No	Ref		Ref	
**History of poor birth outcomes**				
Yes	1.8 (1.3–2.4)	**< 0.001**	-	-
No	Ref		-	-

Abbreviations: human immunodeficiency virus (HIV), adjusted risk ratio (aRR), confidence interval (CI), reference category(ref); Significance level (p ≤ 0.05)

## Discussion

The study found that up to 20.9% of participants experienced adverse birth outcomes. Women that experienced a febrile illness in the current pregnancy, women with a history of poor birth outcomes and primiparous women that had high blood pressure at enrolment had a 1.7, 1.8 and 3.9 times higher risk of an adverse birth outcome respectively. Our findings are comparable to previous studies in Kenya that have reported a prevalence of 2.7% for stillbirths, 0.9% for miscarriage [38], 11.6% for small for gestational age [39], 13% for preterm births [23] and 1.0% for microcephaly [40]. Compared to the rates in the sub-Saharan Africa region, we found similar rates of overall adverse birth outcomes [41], congenital anomalies [42–44] and small for gestational age [45] but higher rates of stillbirths [6, 8] and lower rates of prematurity [7].

Fever was the only modifiable risk factor that was associated with adverse pregnancy in this study. We found that women who had an episode of fever during the current pregnancy had 1.7 times higher risk of an adverse birth outcome compared to those that did not experience fever during pregnancy, and the majority of these women experienced the fever in the third trimester. This finding is corroborated by other studies that report an association between fever and poor birth outcomes [41, 46]. The impact on health following an episode of febrile illness in pregnancy is dependent on the cause, duration and magnitude of temperature elevation, as well as the gestational period in which it occurs, with more severe outcomes when the fever occurs early in pregnancy [47]. Fever poses a risk to the pregnant woman and developing foetus especially when accompanied by an infection [47, 48] which may be transmitted to the foetus in utero and lead to adverse birth outcomes [49]. Fever during the periconceptional period may also disrupt foetal development resulting in central nervous system defects and leading to cognitive and behavioural problems in offspring [49, 50]. A recent study that modelled adverse birth outcomes in Kenya reported an elevated risk of low birth weight, preterm births and neonatal deaths in the coastal sub-counties, which may be an indication of the role of malaria or arboviruses in pregnancy [22]. Thus, mitigation measures such as vector control could prevent these infections that present with fever in pregnancy. Furthermore, screening and detection of fever during ANC clinic visits with appropriate investigations and medical follow-up could help reduce the risk of adverse birth outcomes.

Multiparous women who had a previous history of a poor birth outcome had 1.8 times the risk of poor birth outcome compared to those that did not have a previous history of an adverse birth outcome.This finding is similar to other studies that demonstrated a higher chance or recurrence of an adverse birth outcomes [51, 52]. Possibly, socioeconomic factors, low quality of antenatal care and genetic factors contribute to the recurrence of poor birth outcomes [53]. Understanding modifiable causes of adverse outcomes in previous pregnancies would be important in reducing a recurrence. Conversely, a history of an adverse birth outcome did not significantly influence the likelihood of poor birth outcomes in subsequent pregnancies in a study done among women in a military hospital in India [54]. Women with a previous adverse birth outcome are more likely to seek early preconception and antenatal care and are likely to have more frequent clinic visits in subsequent pregnancies as compared to women without a poor birth history to prevent poor pregnancy outcomes. However, in our study, there was no difference in the start of ANC clinic between women that had a history of poor birth outcomes and those without a history of poor birth outcomes.

Primiparous women that had high blood pressure at enrolment had 3.9 times the risk of adverse birth outcome compared to those that did not have high blood pressure at enrolment. This association aligns with findings from previous research [55, 56]. Elevated blood pressure in pregnancy has previously been linked to poor birth outcomes due to the inability to adapt to the increased metabolic demands and the physiological changes during pregnancy [57, 58]. Therefore, there is need for heightened antenatal surveillance for increased maternal high blood pressure to facilitate early diagnosis, counselling, management and monitoring to improve maternal and fetal outcomes.

We found that HIV status was not significantly associated with having an adverse birth outcome in this study. However, these findings differ from a systematic review that have previously been conducted in sub- Saharan Africa that reported an association between HIV status and adverse birth outcomes [59]. In South Africa, where HIV infection has been associated with adverse birth outcomes [59], up to 96% of HIV positive pregnant women are on ART [60] which is comparable to the 97% PMTCT coverage in Kenya. Despite the high ART rates in both countries, the viral load suppression among pregnant women is 66% in South Africa [60] compared to 98% in Kenya [61]. Our results could be explained by the difference in viral load suppression among pregnant women between the two countries. Our findings support the results of other studies that report a decline in poor birth outcomes among infants born to HIV positive women during the era of increased ART usage [62, 63].

There is a high burden of alcohol and substance use in the coastal region in Kenya compared to other regions [64]. Although the association between substance use and adverse birth outcomes is well documented in other studies [65, 66], we were not able to elicit this association. Often, there are difficulties in eliciting accurate self-reports regarding the use and degree of substance and alcohol use among pregnant women [67], and this may have led to the absence of association.

A strength of this study is the large sample that provided the statistical power to assess the risk factors of adverse birth outcomes and the monthly follow up of the enrolled women to observe pregnancy outcomes. Despite these strengths, there were some limitations. We did not establish the aetiology of fever because the samples were not tested for potential pathogens apart from Zika virus [25] and TORCH (Toxoplasmosis, Rubella, Cytomegalovirus, and Herpes simplex viruses) pathogens (Manuscript in preparation). The women with birth outcome data were older, multiparous, were more likely to be HIV positive and were likely to attend ANC clinic in a private health facility which could have been a potential source of bias in our study which can affect generalization of the findings since there are baseline differences between those that were lost to follow up and those that were not lost to follow up and therefore we would not be able to determine what the estimates would be if they were not lost to follow up The congenital anomalies reported in this study were based on clinical features and some anomalies may have been missed. Lastly, we used a composite outcome and the risk factors for the individual adverse birth outcomes could vary which may mask the associations and also the risk factors could not be pin pointed toa a specific birth outcome.

## Conclusion

We found similar rates of overall adverse birth outcomes, congenital anomalies and small for gestational age but higher rates of stillbirths and lower rates of prematurity compared to the rates in the Sub-Saharan Africa region. The rates of adverse birth outcomes in this study were comparable to other studies conducted in Kenya. Febrile illnesses during current pregnancy and previous history of poor birth outcome were predictive of increased risk of adverse birth outcomes in this population. These risk factors are readily identifiable during ANC and directing targeted interventions including close monitoring to these high-risk groups could lead to improved birth outcomes.

### Electronic supplementary material

Below is the link to the electronic supplementary material.

**Supplementary Material 1: Table 1.** Comparison of characteristics of participants who had delivery outcome data and those without delivery outcome data

## Data Availability

The datasets used and/or analysed during the current study are available from the corresponding author on reasonable request.

## Abbreviations

AIC: Akaike Information Criterion
ANC: Antenatal care
aRR: Adjusted risk ratios
CI: Confidence intervals
HIV: Human Immunodeficiency Virus
IQR: Interquartile range
LMP: Last menstrual period
LRT: Likelihood ratio test
SGA: Small for gestational age
TORCH: Toxoplasmosis, Rubella, Cytomegalovirus, and Herpes simplex viruses
VIF: Variance inflation factor
WHO: World Health organization

## References

[1] Beck S, Wojdyla D, Say L, Betran AP, Merialdi M, Requejo JH (2010). The worldwide incidence of preterm birth: a systematic review of maternal mortality and morbidity. Bull World Health Organ.

[2] Wang H, Bhutta ZA, Coates MM, Coggeshall M, Dandona L, Diallo K (2016). Global, regional, national, and selected subnational levels of stillbirths, neonatal, infant, and under-5 mortality, 1980–2015: a systematic analysis for the global burden of Disease Study 2015. Lancet.

[3] World Health (2016). Standards for improving quality of maternal and newborn care in health facilities. WHO.

[4] Ludvigsson JF, Lu D, Hammarström L, Cnattingius S, Fang F (2018). Small for gestational age and risk of childhood mortality: a Swedish population study. PLoS Med.

[5] Lee ACC, Katz J, Blencowe H, Cousens S, Kozuki N, Vogel JP, et al. National and regional estimates of term and preterm babies born small for gestational age in 138 low-income and middle-income countries in 2010. Lancet Glob Heal. 2013;1(1). 10.1016/S2214-109X(13)70006-8. 10.1016/S2214-109X(13)70006-8 PMC4221634 25103583

[6] Wang H, Abajobir AA, Abate KH, Abbafati C, Abbas KM, Abd-Allah F (2017). Global, regional, and national under-5 mortality, adult mortality, age-specific mortality, and life expectancy, 1970–2016: a systematic analysis for the global burden of Disease Study 2016. Lancet.

[7] Cao G, Liu J, Liu M, Global (2022). Regional, and National Incidence and Mortality of neonatal Preterm Birth, 1990–2019. JAMA Pediatr.

[8] Sitkin NA, Ozgediz D, Donkor P, Farmer DL (2015). Congenital anomalies in low- and middle-income countries:the unborn child of global surgery. World J Surg.

[9] Blencowe H, Cousens S, Oestergaard MZ, Chou D, Moller AB, Narwal R (2012). National, regional, and worldwide estimates of preterm birth rates in the year 2010 with time trends since 1990 for selected countries: a systematic analysis and implications. Lancet.

[10] Lawn JE, Blencowe H, Waiswa P, Amouzou A, Mathers C, Hogan D (2016). Stillbirths: rates, risk factors and potential for progress towards 2030. Lancet.

[11] Moges N, Anley DT, Zemene MA, Adella GA, Solomon Y, Bantie B, et al. Congenital anomalies and risk factors in Africa: a systematic review and meta-analysis. BMJ Paediatr Open. 2023;7(1). 10.1136/bmjpo-2023-002022. 10.1136/bmjpo-2023-002022 PMC10335447 37429669

[12] A Neglected Tragedy. : The global burden of stillbirths - UNICEF DATA.

[13] Nabea GM, Matenjwa Kamau T, Kaburu EW, Kamau TM (2017). The incidence of congenital anomalies among newborns at Pumwani Hospital, Nairobi, Kenya. Int J Heal Sci Res.

[14] Kenya National Bureau of Statistics, ICF Macro. Kenya Demographic Health Surv. 2014;603. 10.3109/03014460.2013.775344.

[15] Kramer MS. The epidemiology of adverse pregnancy outcomes: An overview. J Nutr. 2003;133(5 SUPPL. 1):1592–6. 10.1093/jn/133.5.1592s. 10.1093/jn/133.5.1592S 12730473

[16] Strauss RS (2000). Of those born small for gestational age. J Am Med Assoc.

[17] Asiki G, Baisley K, Newton R, Marions L, Seeley J, Kamali A (2015). Adverse pregnancy outcomes in rural Uganda (1996–2013): trends and associated factors from serial cross sectional surveys. BMC Pregnancy Childbirth.

[18] Lin L, Wei Y, Zhu W, Wang C, Su R, Feng H (2018). Prevalence, risk factors and associated adverse pregnancy outcomes of anaemia in Chinese pregnant women: a multicentre retrospective study. BMC Pregnancy Childbirth.

[19] Saleem S, Tikmani SS, McClure EM, Moore JL, Azam SI, Dhaded SM, et al. Trends and determinants of stillbirth in developing countries: results from the Global Network’s Population-based Birth Registry. Reprod Health. 2018;15(Suppl 1). 10.1186/s12978-018-0526-3. 10.1186/s12978-018-0526-3 PMC6019981 29945647

[20] Ngandu CB, Momberg D, Magan A, Chola L, Norris SA, Said-Mohamed R (2020). The association between household socio-economic status, maternal socio-demographic characteristics and adverse birth and infant growth outcomes in sub-saharan Africa: a systematic review. J Dev Orig Health Dis.

[21] Kagia J (2013). Improving maternal health in Kenya: challenges and strategies for low resource nations. Linacre Q.

[22] Odhiambo JN, Sartorius B. Joint spatio-temporal modelling of adverse pregnancy outcomes sharing common risk factors at sub-county level in Kenya, 2016–2019. BMC Public Health. 2021;1–13. 10.1186/s12889-021-12210-9. 10.1186/s12889-021-12210-9 PMC8719408 34969386

[23] Wagura P, Wasunna A, Laving A, Wamalwa D (2018). Ng’Ang’a P. Prevalence and factors associated with preterm birth at kenyatta national hospital. BMC Pregnancy Childbirth.

[24] Muchemi OM, Echoka E, Makokha A (2015). Factors associated with low birth weight among neonates born at Olkalou district hospital, central region, Kenya. Pan Afr Med J.

[25] Osoro E, Inwani I, Mugo C, Hunsperger E, Verani JR, Omballa V, et al. Prevalence of microcephaly and Zika virus infection in a pregnancy cohort in Kenya, 2017–2019. BMC Med. 2022;1–11. 10.1186/s12916-022-02498-8. 10.1186/s12916-022-02498-8 PMC9470235 36100910

[26] KNBS and ICF (2023). Kenya Demographic and Health Survey 2022. Key indicators Report. Nairobi, Kenya, and Rockville.

[27] WHO recommendations on. antenatal care for a positive pregnancy experience. 28079998

[28] Harris SR (2015). Measuring head circumference: update on infant microcephaly. Can Fam Physician.

[29] Harris PA, Taylor R, Thielke R, Payne J, Gonzalez N, Conde JG (2009). Research electronic data capture (REDCap)-A metadata-driven methodology and workflow process for providing translational research informatics support. J Biomed Inf.

[30] Villar J, Ismail LC, Victora CG, Ohuma EO, Bertino E, Altman DG (2014). International standards for newborn weight, length, and head circumference by gestational age and sex: the newborn cross-sectional study of the INTERGROWTH-21st Project. Lancet.

[31] Kaforau LSK, Tessema GA, Jancey J, Dhamrait G, Bugoro H, Pereira G (2022). Prevalence and risk factors of adverse birth outcomes in the Pacific Island region: a scoping review. Lancet Reg Heal - West Pac.

[32] Price JT, Vwalika B, Rittenhouse KJ, Mwape H, Winston J, Freeman BL, et al. Adverse birth outcomes and their clinical phenotypes in an urban Zambian cohort. Gates Open Res. 2020;3. 10.12688/gatesopenres.13046.2. 10.12688/gatesopenres.13046.1 PMC7047437 32161903

[33] Dash K, Goodacre S, Sutton L (2022). Composite outcomes in clinical prediction modeling: are we trying to predict apples and oranges?. Ann Emerg Med.

[34] Feldkamp ML, Carey JC, Byrne JLB, Krikov S, Botto LD (2017). Etiology and clinical presentation of birth defects: Population based study. BMJ.

[35] Zabransky S. Maternal nutrition. Caring Child Born Small Gestation Age. 2013;25–33. 10.1007/978-1-908517-90-6_3.

[36] Zhang Z (2016). Model building strategy for logistic regression: purposeful selection. Ann Transl Med.

[37] Fagerland MW, Hosmer DW (2012). A generalized Hosmer-Lemeshow goodness-of-fit test for multinomial logistic regression models. Stata J.

[38] Waiswa P, Higgins BV, Mubiri P, Kirumbi L, Butrick E, Merai R (2020). Pregnancy outcomes in facility deliveries in Kenya and Uganda: a large cross-sectional analysis of maternity registers illuminating opportunities for mortality prevention. PLoS ONE.

[39] Simiyu DE (2004). Morbidity and mortality of low Birth Weight infants in the New Born Unit of Kenyatta National Hospital, Nairobi. East Afr Med J.

[40] Barsosio HC, Nyamwaya DK, Gitonga JN, Karanja HK, Omuoyo DO, Kamau E (2019). Congenital microcephaly unrelated to flavivirus exposure in coastal Kenya [version 1; peer review: 2 approved, 1 approved with reservations]. Wellcome Open Res.

[41] Tamirat KS, Sisay MM, Tesema GA, Tessema ZT (2021). Determinants of adverse birth outcome in Sub-saharan Africa: analysis of recent demographic and health surveys. BMC Public Health.

[42] Adane F, Afework M, Seyoum G, Gebrie A (2020). Prevalence and associated factors of birth defects among newborns in sub-saharan African countries: a systematic review and meta-analysis. Pan Afr Med J.

[43] Mashuda F, Zuechner A, Chalya PL, Kidenya BR, Manyama M (2014). Pattern and factors associated with congenital anomalies among young infants admitted at Bugando medical centre, Mwanza, Tanzania. BMC Res Notes.

[44] Uba AF, Igun GO, Kidmas AT, Chirdan LB (2004). Prevalence of umbilical hernia in a private school admission-seeking Nigerian children. Niger Postgrad Med J.

[45] Lee ACC, Kozuki N, Cousens S, Stevens GA, Blencowe H, Silveira MF, et al. Estimates of burden and consequences of infants born small for gestational age in low and middle income countries with INTERGROWTH-21 St standard: analysis of CHERG datasets. BMJ. 2017;358. 10.1136/bmj.j3677. 10.1136/bmj.j3677 PMC5558898 28819030

[46] Sass L, Urhoj SK, Kjærgaard J, Dreier JW, Strandberg-Larsen K, Nybo Andersen AM (2017). Fever in pregnancy and the risk of congenital malformations: a cohort study. BMC Pregnancy Childbirth.

[47] More VS (2017). Fever in pregnancy and its maternal and fetal outcomes. Int J Reprod Contracept Obstet Gynecol.

[48] Sultan S, Parihar R, Badkur P (2020). Study on pyrexia in pregnancy and labour with special emphasis on fetomaternal outcome. Int J Reprod Contracept Obstet Gynecol.

[49] Sappenfield E, Jamieson DJ, Kourtis AP (2013). Pregnancy and susceptibility to infectious diseases. Infect Dis Obstet Gynecol.

[50] Kerr SM, Parker SE, Mitchell AA, Tinker SC, Werler MM (2017). Periconceptional maternal fever, folic acid intake, and the risk for neural tube defects. Ann Epidemiol.

[51] Saroj Chakraborty S, Galla X, Cheng J-Y, Yeo B, Mell V, Singh BS, Yeoh P, Saha AV, Mathew (2017). Matam Vijay-Kumar and BJ. HHS Public Access. Physiol Behav.

[52] Su D, Samson K, Garg A, Hanson C, Anderson Berry AL, Lin G (2018). Birth history as a predictor of adverse birth outcomes: evidence from state vital statistics data. Prev Med Rep.

[53] Sclowitz IKT, Santos IS, Domingues MR, Matijasevich A, Barros AJD. Prognostic factors for low birthweight repetition in successive pregnancies: a cohort study. BMC Pregnancy Childbirth. 2013;13. 10.1186/1471-2393-13-20. 10.1186/1471-2393-13-20 PMC3558483 23342985

[54] Singh G, Sidhu K. Bad obstetric history: a prospective study. Med J Armed Forces India 66(2):117–20. 10.1016/S0377-1237(10)80121-2. 10.1016/S0377-1237(10)80121-2 PMC4920907 27365723

[55] Jin M, Liu X, Liu X, Wu Y, Zhang Y, Zhang L (2024). Association of pre-/early pregnancy high blood pressure and pregnancy outcomes: a systemic review and meta-analysis. J Matern Fetal Neonatal Med.

[56] Bramham K, Parnell B, Nelson-Piercy C, Seed PT, Poston L, Chappell LC (2014). Chronic hypertension and pregnancy outcomes: systematic review and meta-analysis. BMJ.

[57] Macdonald-wallis C, Lawlor DA, Fraser A, May M, Scott M (2012). Blood pressure change in normotensive, gestational hypertensive, preeclamptic and essential hypertensive pregnancies. Hypertension.

[58] Zhu J, Zhang J, Ng MJ, Chern B, Yeo GSH, Tan KH (2019). Angiogenic factors during pregnancy in Asian women with elevated blood pressure in early pregnancy and the risk of preeclampsia: a longitudinal cohort study. BMJ Open.

[59] Murray C, Portwood C, Sexton H, Kumarendran M, Brandon Z, Kirtley S (2023). Adverse perinatal outcomes attributable to HIV in sub-saharan Africa from 1990 to 2020: systematic review and meta-analyses. Commun Med.

[60] Woldesenbet S, Cheyip M, Lombard C, Manda S, Ayalew K, Kufa T (2022). Progress towards the UNAIDS 95-95-95 targets among pregnant women in South Africa: results from the 2017 and 2019 national antenatal HIV sentinel surveys. PLoS ONE.

[61] Bond E. Innovations And Impact Toward The Elimination Of Mother-To-Child Transmission In Kenya. 2019.

[62] Macdonald EM, Ng R, Bayoumi AM, Raboud J, Brophy J, Masinde K, irene (2015). Living with HIV: a Population-based study. J Obstet Gynaecol Can.

[63] Koss CA, Natureeba P, Plenty A, Luwedde F, Mwesigwa J, Ades V (2014). Risk factors for preterm birth among HIV-infected pregnant Ugandan women randomized to lopinavir/ritonavir-or efavirenz-based antiretroviral therapy. J Acquir Immune Defic Syndr.

[64] Weldon K (2013). An analysis of drug abuse along the coastal region of Kenya. Int NGO J.

[65] Quesada O, Gotman N, Howell HB, Funai EF, Rounsaville BJ, Yonkers KA (2012). Prenatal hazardous substance use and adverse birth outcomes. J Matern Neonatal Med.

[66] Dejong K, Olyaei A, Lo JO (2019). Alcohol use in pregnancy. Clin Obstet Gynecol.

[67] Aylin P, Bennett P, Bottle A, Brett S, Sodhi V, Rivers A (2016). Estimating the risk of adverse birth outcomes in pregnant women undergoing non-obstetric surgery using routinely collected NHS data: an observational study. Heal Serv Deliv Res.

