# Supplementary material for: Risk factors of adverse birth outcomes among a cohort of pregnant women in Coastal Kenya, 2017–2019
Source: BMC Pregnancy Childbirth. 2024 Feb 12;24:127. doi: 10.1186/s12884-024-06320-6 (PMC10860222; doi:10.1186/s12884-024-06320-6)
Supplement: Supplementary file 1 — Supplementary Material 1: Table 1. Comparison of characteristics of participants who had delivery outcome data and those without delivery outcome data [file 12884_2024_6320_MOESM1_ESM.pdf]

Risk factors of adverse birth outcomes among a cohort of pregnant women in Coastal Kenya, 2017-2019

Harriet Mirieri[1]*, Ruth Nduati[2], Jeanette Dawa[1], Lydia Okutoyi[3], Eric Osoro[1,4], Cyrus Mugo[5], Dalton Wamalwa[2], Hafsa Jin[6], Dufton Mwaengo[7], Nancy Otieno[8], Doris Marwanga[1], Mufida Shabibi[9], Peninah Munyua[10] John Kinuthia[5], Erin Clancey[4], Marc-Alain Widdowson[10,12¶], M. Kariuki Njenga[1,4], Jennifer R. Verani[10], Irene Inwani[11]

Supplementary table 1: Comparison of characteristics of participants who had delivery outcome data and those without delivery outcome data.

Characteristic	N	Total n (%)	Delivery outcome: With delivery outcome data (n=1916) n (%)	Delivery outcome: Without delivery outcome data (n=396) n (%)	OR (95% CI)	p-value
Age, years median (IQR)		28 (24-32)	28(24-33)	27(23-30)	1.1 (1.1-1.2)	<0.001*
**Health facility type**	2312					
Public		1558 (67.4)	1262 (65.9)	296 (74.8)	Ref	
Private		754 (32.6)	654 (34.1)	100 (25.3)	1.5 (1.2-1.9)	0.001*
**Education**	2278					
Primary		592 (26.0)	490 (26.0)	102 (26.0)	Ref	
Secondary		1016 (44.6)	857 (45.5)	159 (40.4)	1.1(0.8-1.5)	0.407
College (Middle level)		670 (29.4)	538 (28.5)	132 (33.6)	0.8(0.6-1.1)	0.260
**Employment**	2312					
Employed		898(38.9)	754(39.4)	144(36.6)	1.2(0.9-1.5)	0.161
Self employed		339(14.7)	285(14.9)	54(13.6)	1.2(0.9-1.7)	0.294
Unemployed		1074(46.5)	876(45.7)	198(50.0)	Ref	
**Marital status**	2312					
Married		2040 (88.2)	1699 (88.7)	341 (86.1)	1.3(0.9-1.7)	0.150
Single		272 (11.8)	217 (11.3)	55 (13.4)	Ref	
**Chronic illness**	2312					
Asthma		83 (3.6)	68 (3.6)	15 (3.8)	1.1(0.6-1.9)	0.806
Diabetes		16 (0.7)	13 (0.7)	3 (0.7)	1.1(0.3-3.9)	0.865

Epilepsy		7 (0.3)	6 (0.3)	1 (0.3)	1.2(0.1-10.4)	0.840
Hypertension		44 (1.9)	36 (1.9)	8 (2.0)	0.9(0.4-2.0)	0.852
**Lifestyle factors**	2312					
Substance use		87(3.8)	75(3.9)	12(3.0)	1.3(0.7-2.4)	0.401
**HIV status**	2160					
HIV positive		343 (15.9)	308 (17.2)	35 (9.9)	1.9(1.3-2.7)	0.001*
On HAART	336	310(92.3)	278(91.8)	32(97.0)	0.3(0.04-2.6)	0.308
**Syphilis status**	2072					
Positive		29(1.4)	26(1.5)	3(0.9)	1.7(0.5-5.8)	0.368
**Obstetric factors**						
Multiparity	2310	1620 (70.1)	1367 (71.4)	253 (63.9)	1.4(1.1-1.7)	0.003*
History of preeclampsia	1613	1613	94(6.9)	16(6.3)	1.1(0.6-1.9)	0.734
History of premature birth	1611	96(5.9)	75(5.5)	21(8.3)	0.6(0.4-1.1)	0.089
History of low birth weight	1610	120(7.5)	101(7.4)	19(7.6)	0.9(0.6-1.6)	0.908
**County of residence**	2312					
Within Mombasa County		2237(96.7)	1858(96.9)	379(95.7)	1.4 (0.8-2.5)	0.198
Outside Mombasa County		75(3.1)	58(3.1)	17(4.3)	Ref	

***Variables significant at p value ≤0.05**

Abbreviations: Human immunodeficiency virus (HIV), odds ratio (OR), interquartile range (IQR), antiretroviral therapy (HAART)
